# M1 cholinergic signaling in the brain modulates cytokine levels and splenic cell sub-phenotypes following cecal ligation and puncture

**DOI:** 10.1186/s10020-024-00787-x

**Published:** 2024-02-05

**Authors:** Mabel N. Abraham, Ana Nedeljkovic-Kurepa, Tiago D. Fernandes, Omar Yaipen, Mariana R. Brewer, Daniel E. Leisman, Matthew D. Taylor, Clifford S. Deutschman

**Affiliations:** 1 Department of Pediatrics, Cohen Children’s Medical Center, Northwell Health, New Hyde Park, New York, USA; 2 Sepsis Research Laboratories, The Feinstein Institutes for Medical Research, Northwell Health, Room 3140, 350 Community Drive, Manhasset, NY 11030 USA; 3 Zucker School of Medicine at Hofstra/Northwell, Hempstead, NY USA; 4 Department of Medicine, Massachusetts General Hospital, Boston, USA

**Keywords:** Sepsis, Sepsis-3, Organ dysfunction, Cecal ligation and puncture, Basal forebrain cholinergic system, Muscarinic receptors, Xanomeline, Immune dysfunction

## Abstract

**Background:**

The contribution of the central nervous system to sepsis pathobiology is incompletely understood. In previous studies, administration of endotoxin to mice decreased activity of the vagus anti-inflammatory reflex. Treatment with the centrally-acting M1 muscarinic acetylcholine (ACh) receptor (M1AChR) attenuated this endotoxin-mediated change. We hypothesize that decreased M1AChR-mediated activity contributes to inflammation following cecal ligation and puncture (CLP), a mouse model of sepsis.

**Methods:**

In male C57Bl/6 mice, we quantified basal forebrain cholinergic activity (immunostaining), hippocampal neuronal activity, serum cytokine/chemokine levels (ELISA) and splenic cell subtypes (flow cytometry) at baseline, following CLP and following CLP in mice also treated with the M1AChR agonist xanomeline.

**Results:**

At 48 h. post-CLP, activity in basal forebrain cells expressing choline acetyltransferase (ChAT) was half of that observed at baseline. Lower activity was also noted in the hippocampus, which contains projections from ChAT-expressing basal forebrain neurons. Serum levels of TNFα, IL-1β, MIP-1α, IL-6, KC and G-CSF were higher post-CLP than at baseline. Post-CLP numbers of splenic macrophages and inflammatory monocytes, TNFα^+^ and ILβ^+^ neutrophils and ILβ^+^ monocytes were higher than baseline while numbers of central Dendritic Cells (cDCs), CD4^+^ and CD8^+^ T cells were lower. When, following CLP, mice were treated with xanomeline activity in basal forebrain ChAT-expressing neurons and in the hippocampus was significantly higher than in untreated animals. Post-CLP serum concentrations of TNFα, IL-1β, and MIP-1α, but not of IL-6, KC and G-CSF, were significantly lower in xanomeline-treated mice than in untreated mice. Post-CLP numbers of splenic neutrophils, macrophages, inflammatory monocytes and TNFα^+^ neutrophils also were lower in xanomeline-treated mice than in untreated animals. Percentages of IL-1β^+^ neutrophils, IL-1β^+^ monocytes, cDCs, CD4^**+**^ T cells and CD8^**+**^ T cells were similar in xanomeline—treated and untreated post-CLP mice.

**Conclusion:**

Our findings indicate that M1AChR-mediated responses modulate CLP-induced alterations in serum levels of some, but not all, cytokines/chemokines and affected splenic immune response phenotypes.

**Supplementary Information:**

The online version contains supplementary material available at 10.1186/s10020-024-00787-x.

## Background

Sepsis is defined as life-threatening organ dysfunction caused by a dysregulated host response to infection (Singer et al. 2016). Studies have suggested that central cholinergic (ACh) dysfunction contributes to sepsis pathobiology (Zaghloul et al. 2017; Santos-Junior et al. 2018; Bricher Choque et al. 2021). In particular, recent studies have demonstrated decreased activity in the basal forebrain cholinergic system of mice subjected to cecal ligation and puncture (CLP, the most commonly used animal model of sepsis (Zaghloul et al. 2017; Yin et al. 2023; Zhai et al. 2017; Osuchowski et al. 2018). This system, which is involved in altered executive functions in neurodegenerative disorders (Berlot et al. 2022; Dwomoh et al. 2022; Scarpa et al. 2020), also contributes to immune dysfunction (Bricher Choque et al. 2021; Zhai et al. 2017; Lehner et al. 2019; Munyaka et al. 2014), one of the defining characteristics of sepsis (Singer et al. 2016). In particular, Zhai et al. used optogenetic stimulation to activate basal forebrain neurons that express choline acetyltransferase (ChAT, the enzyme that catalyzes the rate-limiting step in ACh biosynthesis) at very early time points post-CLP (Zhai et al. 2017). Optogenetic activation increased activity at several loci in the brainstem and decreased serum levels of TNFα and IL-6 via a mechanism that involved the vagus nerve and the spleen. Interestingly, Rosas-Ballina et al. used xanomeline, a centrally acting M1AChR agonist, to reverse the effects of lipopolysaccharide (LPS) on the relative abundance of several splenic cell subsets and on induced cytokine production by splenic cells (Rosas-Ballina et al. 2015). In the same study, xanomeline improved survival following CLP (Rosas-Ballina et al. 2015). Immune abnormalities are a key component of the dysregulated host response that causes organ dysfunction, the defining characteristic of sepsis (Singer et al. 2016). These two studies led us to test the hypothesis that altered central M1AChR-mediated activity contributes to CLP-induced effects on the release of an extended set of cytokines and chemokines (primary outcome) and alters the relative distribution of splenic cell subtypes (secondary outcome).

## Methods

Experiments were conducted on 12–16-week-old male C57Bl/6 mice (Jackson Labs, Bar Harbor ME). Following historical precedent, males alone were used because survival from CLP in females is substantially higher than in males. Animals were housed in a veterinarian—supervised facility and were acclimated for a minimum of one week prior to use. All studies were approved by the Feinstein Institute IACUC (2017-013 Term I & II) and conformed to ARRIVE guidelines.

### Cecal ligation and puncture

CLP was performed under isoflurane anesthesia using two 22—gauge punctures in a fully ligated cecum as previously described (Abraham et al. 2018). Animals were resuscitated with a subcutaneous (SQ) injection of 50 mL/kg of sterile saline and received 0.5 mg/kg of imipenim/cilastatin SQ at the end of surgery and at 24 h. post-procedure. As in previous studies, mortality at 48 h. post-CLP was about 50% (Abraham et al. 2018; Abcejo et al. 2009). Blood was obtained via cheek bleed and mice were then euthanized by decapitation. Organs, including brains, were harvested with brain tissues fixed in 4% paraformaldehyde. Previous experience has demonstrated that, by about 6 h. post-procedure, the variables of interest in unoperated mice and sham-operated controls were identical (Abcejo et al. 2009; Haaxma et al. 2003). Therefore, we have discontinued the use of sham-operated controls. Importantly, our findings are consistent with studies on the orexinergic effects of anesthetics, which have disappated within the 6 h. timeframe (Kelz et al. 2008).

### Xanomeline administration

Xanomeline (5 mg/kg in 0.2 ml saline) was administered intraperitoneally at the time of CLP and at 24and 47 h. post-CLP for a total dose of 15 mg/kg. Mice were euthanized one hour after the final dose. We elected not to study animals that received vehicle only following CLP because multiple studies have demonstrated that injection of 0.2 ml of saline did not affect post-CLP parameters (Osuchowski et al. 2018; Deutschman et al. 2013; Leisman et al. 2022).

### Measurements of serum cytokines

Serum was separated from blood by centrifugation, aliquoted and frozen at -80ºC until used. Levels of TNFα, IL-1β, IL-6, MIP-1α, KC, and G-CSF were determined using a multiplex ELISA (Eve Technologies, Calgary, Alberta, Canada).

### Leukocyte isolation

As previously described (Taylor et al. 2020a), spleens harvested post-euthanasia were immediately digested with DNAse (100 µg/mL) and Collagenase A (1 mg/mL) in complete media for 30 min at 37 °C. Cells were passed through a 70 µm filter and resuspended. Red blood cells were lysed, white cells were counted using a Countess II Automated Cell Counter (ThermoFisher, Waltham, MA) and spleen cells were analyzed using flow cytometry. A minimum of 2 × 10^6^ events were analyzed for each sample.

### Cytokine production assays

Single cell suspensions were stimulated with LPS (500 ng/ml) for 3 h in the presence of Brefeldin A as previously described (Taylor et al. 2022). Concurrently prepared cell suspensions without stimulation served as controls for background production.

### Flow cytometry

Single-cell suspensions were stained for flow cytometric analysis with LIVE/DEAD fixable viability dye (Life Technologies) and the following antibodies: CD90.2, CD8a, CD4, Ly6C, CD11c, Ly6G, MHCII, IL1β, and TNFα (antibody details—Additional file 2: Table S1). All flow cytometric analysis was performed on a BD LSR Fortessa 16-color cell analyzer and analyzed using FlowJo software version 10 (BD Bioscience, San Jose, CA). We used fluorescence minus one controls to validate all antibodies. Stimulation assays were performed alongside and compared to unstimulated controls to ensure only true cytokine producing cells were gated. Gating strategies are included in Additional file 1: Fig. S1.

### Brain harvesting, preparation and staining

Brains were fixed with 4% paraformaldehyde for 48 h, immersed in 30% sucrose, embedded into optimal temperature cutting compound and sliced to yield 10 µmsections; sagittal sections were examined in studies of basal forebrain, coronal sections were used to evaluate staining in the hippocampus. Sections that contained basal forebrain were identified using the Allen Brain Atlas based on characteristic structure and location. Basal forebrain sections were co-immunostained with antibodies to choline acetytransferase (ChAT, goat anti-ChAT, 1:100, EMD Millipore Corp, Burlington VT),and c-Fos (rabbit polyclonal, 1:500, Cell Signaling, Danvers, MA). Secondary antibodies were donkey anti-goat conjugated to Alexa 488 for ChAT, donkey anti-rabbit conjugated to Alexa 594 for c-Fos, and donkey anti-mouse Alexa 488 for c-Fos). Immunofluorescence intensity was determined in 10 non-contiguous 40x-powered fields per slide, 1–2 slides/animals.

The fraction of activated cells was determined as.

$$\frac{{\rm Tota\,l number\, of\, cells\, expressing\, both\, ChAT\, and\, c-fos}}{{\rm Number\, of\, cells\, expressing\, both\, ChAT\, and\, c-fos} + {\rm number\, of\, cells\, expressing \,ChAT\, only}}$$

This fraction was reported as percentage.

### Statistics

To compare treatment groups, we used one-way ANOVA with Tukey–Kramer corrections for multiple comparisons. Prior to analysis, data distributions were evaluated graphically. Several measurements were found to take right-skewed distributions. In these cases, a natural logarithm transformation was accordingly applied to these data prior to analyzing group differences, as is common practice for beta-distributed data (Harrell 2015) (See Additional file 1: Figure S3. for representative residual and Q-Q plots). Differences were considered statistically significant when P < 0.05. In all analyses, the unit-of-analysis was the individual animal. All analyses were performed using Prism version 10.1.1 (GraphPad Software, Boston, MA).

## Results

### Basal forebrain cholinergic activity is lower following CLP than at baseline

**Fig. 1:**
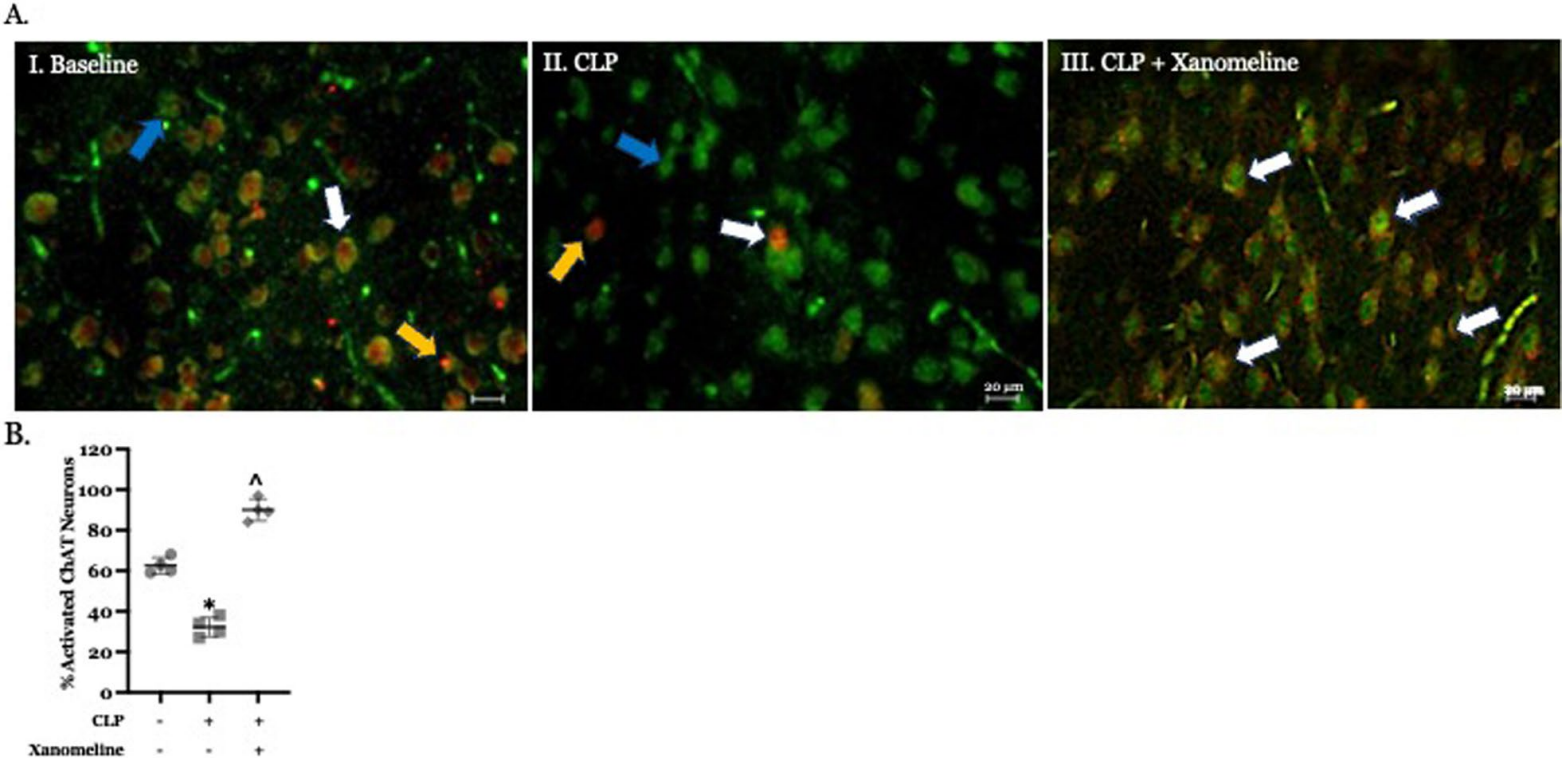
Effects of CLP and CLP + xanomeline treatment on activity in the basal forebrain cholinergic system. C57Bl6 mice, euthanized 48 h. post CLP. **A** Representative immunostained sagittal sections. Scanned and imaged on Leica DMI 4000b using Allen Brain Atlas (P56 Image 21) at 20 × magnification. Green stain (fluorescence from Alexa 488, identified by blue arrows)—Choline Acetyl Transferase (ChAT)—containing neurons; Red stain (fluorescence from Alexa 594, indicated by yellow arrows)—c-Fos expressing neurons; neurons expressing both ChAT and c-Fos indicated by white arrows. **B** Quantification of active neurons that expressed ChAT. Immunofluorescent activity of ChAT (Alexa 488) and c-Fos (Alexa 594) determined in 10 non-contiguous 20x-powered fields per slide, 1–2 slides/animal. Mean value for each animal indicated by closed circle (T_0_/baseline), closed square (48 h. post CLP) or closed diamond (xanomeline treatment of mice studied 48 h. post-CLP). N = 4 mice for each set of measurements. Long horizontal line—mean of measurements in all four animals; lighter lines—± standard deviation. Significance determined using one-way ANOVA with Tukey–Kramer correction, P < 0.05. * = significantly different from T_0_. ^= significantly different from value at 48 h. post-CLP without xanomeline

Previous studies have demonstrated that LPS administration reduced basal forebrain cholinergic activity (Yin et al. 2023; Rosas-Ballina et al. 2015). However, the inflammatory responses to LPS and CLP differ substantially. These differences might extend to activity in specific brain regions. We therefore sought to examine the effects of CLP on the activity of basal forebrain neurons expressing ChAT. Our studies revealed that, at 48 h. post-CLP, basal forebrain co-localization of ChAT and c-Fos, a marker for recent neural depolarization, was significantly lower than that observed at baseline (T_0_) (Fig. 1A, B). At the same post CLP time point, co-localization of ChAT and c-Fos in mice treated with the M1AChR agonist xanomeline was significantly higher than that observed in untreated animals, as well as in unoperated controls (Fig. 1A, B). These findings suggest that the CLP-induced decrease in basal forebrain cholinergic activity is driven, at least in part, by a difference in M1AChR-mediated activity.

### Post-CLP activity in the hippocampus is lower than baseline and is altered by the M1AChR agonist xanomeline

**Fig. 2:**
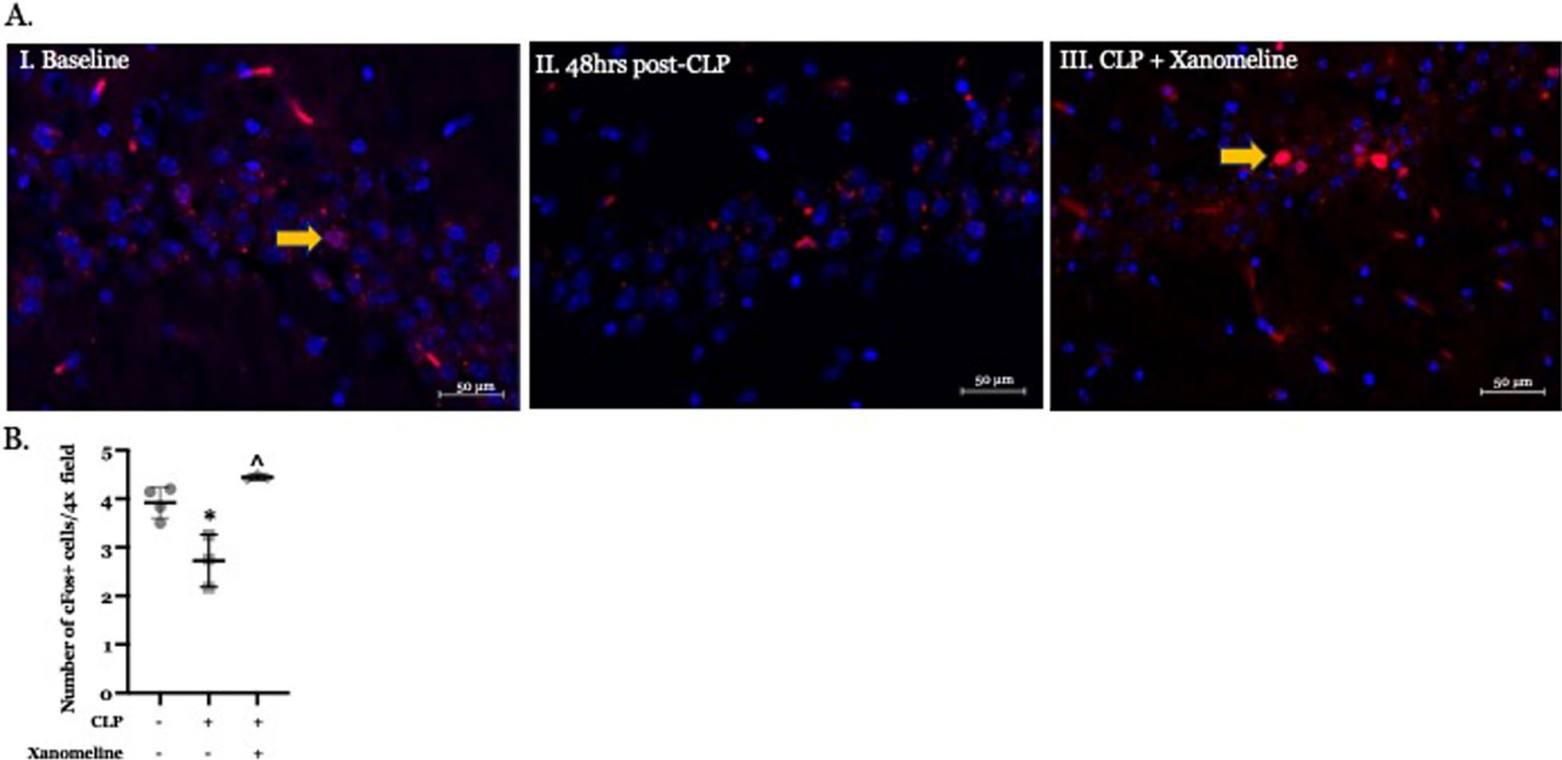
Effects of CLP and CLP + xanomeline treatment on activity in the hippocampus. C57Bl6 mice, euthanized 48 h. post CLP. **A** Representative immunostained coronal sections. Scanned and imaged on Zeiss LSM 880 at 40 × magnification. Red stain (fluorescence from Alexa 594, indicated by yellow arrows)—c-Fos expressing cells. Blue stain (DAPI)—cell nuclei. **B** Quantification of active hippocampal cells. Immunofluorescent activity of c-Fos (Alexa 594) determined in 6–7 non-contiguous 40x-powered fields per slide, 1–2 slides/animal. Mean value for each animal indicated by closed circle (T_0_/baseline), closed square (48 h. post CLP) or closed diamond (xanomeline treatment of mice studied 48 Hrs. post-CLP). N = 3–4 mice for each set of measurements. Long horizontal line—mean of measurements in all 3–4 animals; lighter lines—± standard deviation. Significance determined using one-way ANOVA with Tukey–Kramer correction, P < 0.05. *= significantly different from T_0_. ^= significantly different from value at 48 h. post-CLP without xanomeline

Muscarinic AChRs are activated by ACh-secreting basal forebrain neurons. These receptors have been identified in several different regions of the brain, including but not limited to the cortex, thalamus, hippocampus, neostriatum and basal forebrain (Fernandez de Sevilla et al. 2021; Sarter and Lustig 2020). Xanomeline directly activates M1 and M4AChRs (Shannon et al. 1994). M1 receptors are predominantly post-synaptic and increase neuronal activity while pre-synaptic M4 receptors limit ACh release. Thus, a xanomeline-induced increase in neuronal activation likely results from M1 stimulation while a decrease should reflect M4-mediated effects. We therefore used immunostaining with an antibody to c-Fos to compare activity at T_0_ with the effects of CLP and CLP + xanomeline on activity in several brain regions. Baseline expression of c-Fos was most pronounced in the hippocampus, a finding consistent with previous reports (Berlot et al. 2022). At 48 h post-CLP c-Fos expression in this region was significantly lower than at T_0_ (Fig. 2A, B). In contrast, c-Fos expression following CLP + xanomeline was significantly higher than those observed in untreated mice and could not be distinguished from activity observed at T_0_. Therefore, our findings suggest that CLP-induced decreases in hippocampal neuronal activity result, in part, from an attenuation of M1AChR stimulation.

### Reduced central M1AChR-mediated activity contributes to CLP-induced elevation in serum levels of some, but not all, cytokines

**Fig. 3:**
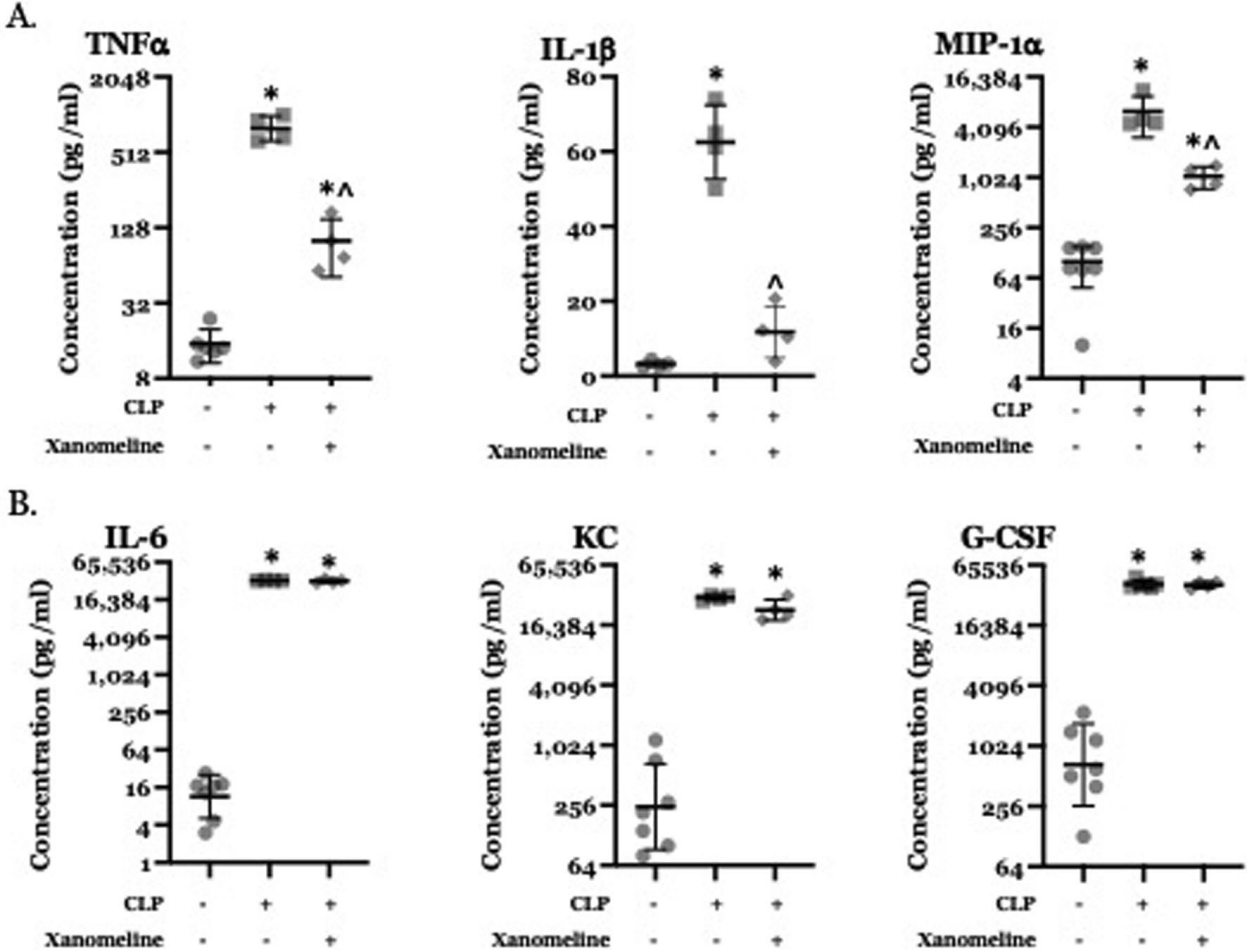
Effects of CLP and CLP + xanomeline treatment on serum concentrations of selected cytokines. C57Bl6 mice, euthanized 48 h. post CLP. Levels (pg/ml) determined using multiplex ELISA (Eve Technologies, Calgary, Alberta, Canada). Mean value for each animal indicated by closed circle (T_0_/baseline), closed square (48 h. post-CLP) or closed diamond (xanomeline treatment of mice studied 48 h. post-CLP). Linear Y-axis in IL-1b plot, log_2_ Y-axes in all other plots. Long horizontal lines—mean value (arithmetic for IL-1β, geometric for others); error bars—± standard deviation (arithmetic for IL-1β, geometric for others). For TNFα, MIP-1α, IL-6, KC, and G-CSF, data were ln-transformed for analysis. Statistical comparisons between groups with ordinary one-way ANOVA with Tukey–Kramer correction (P < 0.05) applied to results. *= significantly different from value at T_0_; ^= significantly different from value at 48 h. post-CLP without xanomeline. **A** Serum concentrations of TNFα, IL-1β and MIP-1α. N = 4–7. **B** Serum concentrations of IL-6, KC and G-CSF. N = 4–7

Previous studies demonstrated that serum concentrations of TNFα and IL-6 were increased by LPS administration (Rosas-Ballina et al. 2015). Levels were reduced in mice pre-treated with the centrally acting M1AChR agonist xanomeline; this effect was not present in animals receiving saline (Rosas-Ballina et al. 2015). Post-CLP levels of these two cytokines were also higher than at T_0_; optogenetic stimulation of ChAT-expressing basal forebrain neurons lowered TNFα and IL-6 levels (Zhai et al. 2017). We therefore examined the effects of xanomeline administration on CLP-induced elevations of serum levels of TNFα, IL-6 and several other cytokines and chemokines. Data are presented in Fig. 3. At 48 h. post-CLP, serum levels of TNFα, IL-1β, MIP-1α, IL-6, KC and G-CSF were higher than at T_0_ (Fig. 3A, B). Following treatment with xanomeline, serum TNFα, IL-1β and MIP-1α levels were lower than those observed following CLP alone and were indistinguishable from levels seen prior to CLP (Fig. 3A). In contrast, levels of IL-6, KC and G-CSF following CLP + xanomeline were not statistically distinguishable from levels observed following CLP alone (Fig. 3B). These findings indicate that M1AChR-mediated responses modulate serum levels of some, but not all, cytokines.

### Central M1AChR-mediated responses modulate CLP-induced differences in the distribution of splenic cell subgroups

**Fig. 4:**
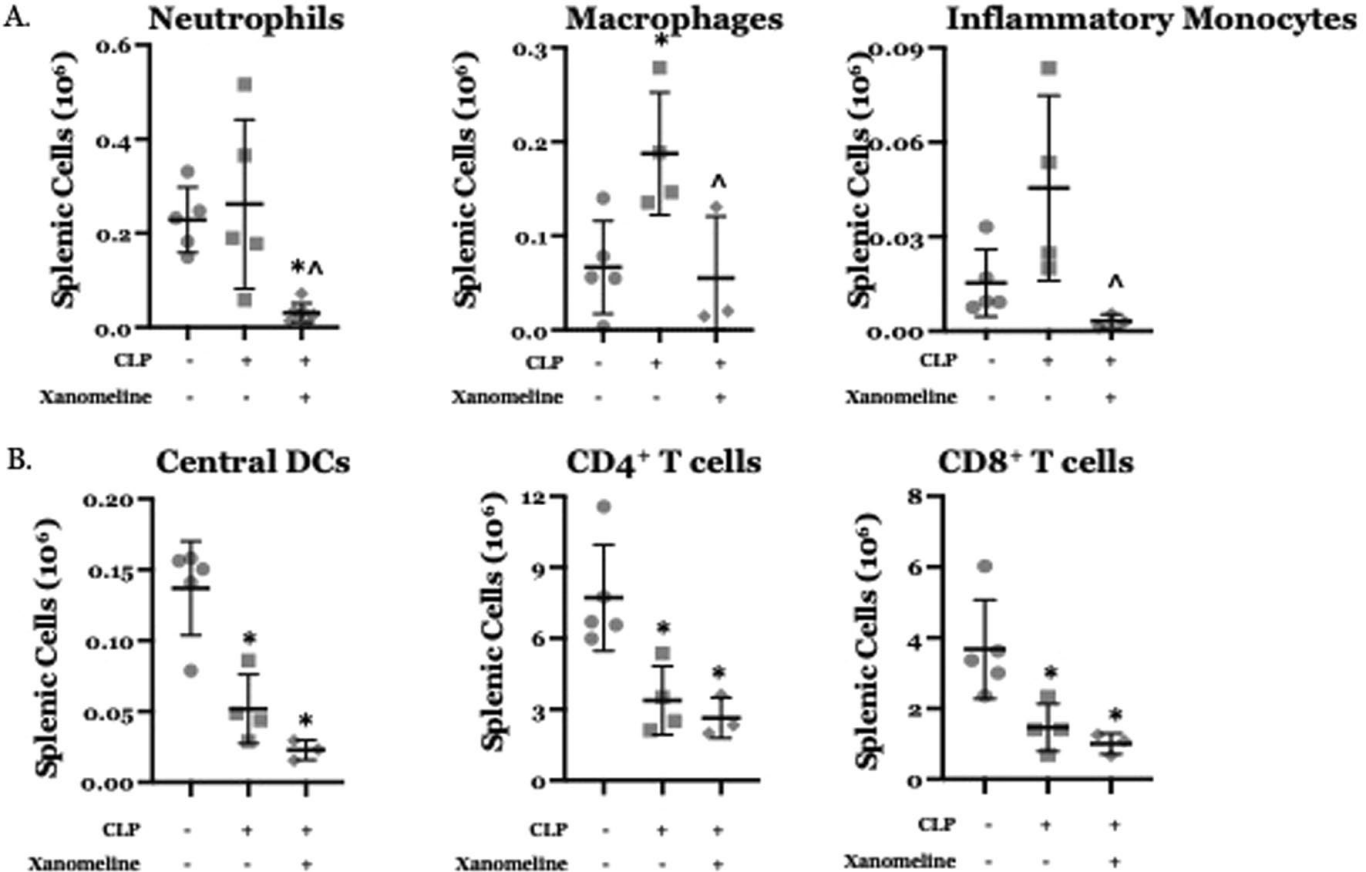
Effects of CLP and CLP + xanomeline treatment on splenic cell subgroups. C57Bl6 mice, euthanized 48 h. post CLP. Cell types/cytokines identified using flow cytometry. Mean value for each animal indicated by closed circle (T_0_/baseline), closed square (48 h. post CLP) or closed diamond (xanomeline treatment of mice studied 48 Hrs. post-CLP). Long horizontal lines—mean value; error bars—± standard deviation. Significance determined using one-way ANOVA with Tukey–Kramer correction, P < 0.05. *= significantly different from value at T_0_; ^= significantly different from value at 48 h. post-CLP without xanomeline. N = 3–7. **A** Numbers of Neutrophils, Macrophages and Inflammatory Monocytes. **B** Numbers of Central DCs, CD4^+^ T cells and CD8^+^ T cells

Previous work has demonstrated that the effects of LPS/inflammation on the relative proportions and activation states of both innate and adaptive immune cells are influenced by cholinergic neural input (Bricher Choque et al. 2021; Lehner et al. 2019; Munyaka et al. 2014; Rosas-Ballina et al. 2015; Taylor et al. 2020a, 2022; Mina-Osorio et al. 2012; Huston et al. 2009). To examine the contribution of M1AChR-mediated signaling to CLP-induced differences in immune cell phenotypes we harvested splenic cells at 48 h. post CLP from mice treated with either vehicle or xanomeline. Using flow cytometry, we examined the abundance of these cells and their ability to mount a cytokine response to ex vivo LPS stimulation. At 48 h. post-CLP, the absolute numbers of splenic macrophages (CD11b^+^/Ly6G^−^/MHCII^−^/CD64^+^/Ly6C^mid^) and inflammatory monocytes (CD11b^+^/Ly6G^−^/MHCII^−^/CD64^−^/Ly6C^hi^) were both higher than at baseline (Fig. 4A). When post-CLP mice were treated with xanomeline, the numbers of neutrophils (CD11b^+^/Ly6G^+^), macrophages and inflammatory monocytes were lower than following CLP alone and could not be distinguished from numbers observed at baseline (Fig. 4A). Post-CLP the numbers of central dendritic cells (cDCs, CD11c^+^/MHCII^+^) and of CD4^+^ (CD90^+^/CD4^+^) and CD8^+^ (CD90^+^/CD8^+^) T cells were lower than baseline (Fig. 4B). However, xanomeline did not affect these observed differences, suggesting that the effects of CLP on cDCs and T cell numbers are M1AChR -independent. These data are consistent with previous work demonstrating that CLP profoundly affected the numbers of splenic T cells (Taylor et al. 2020b) and dendritic cells (Hotchkiss et al. 2002).

**Fig. 5:**
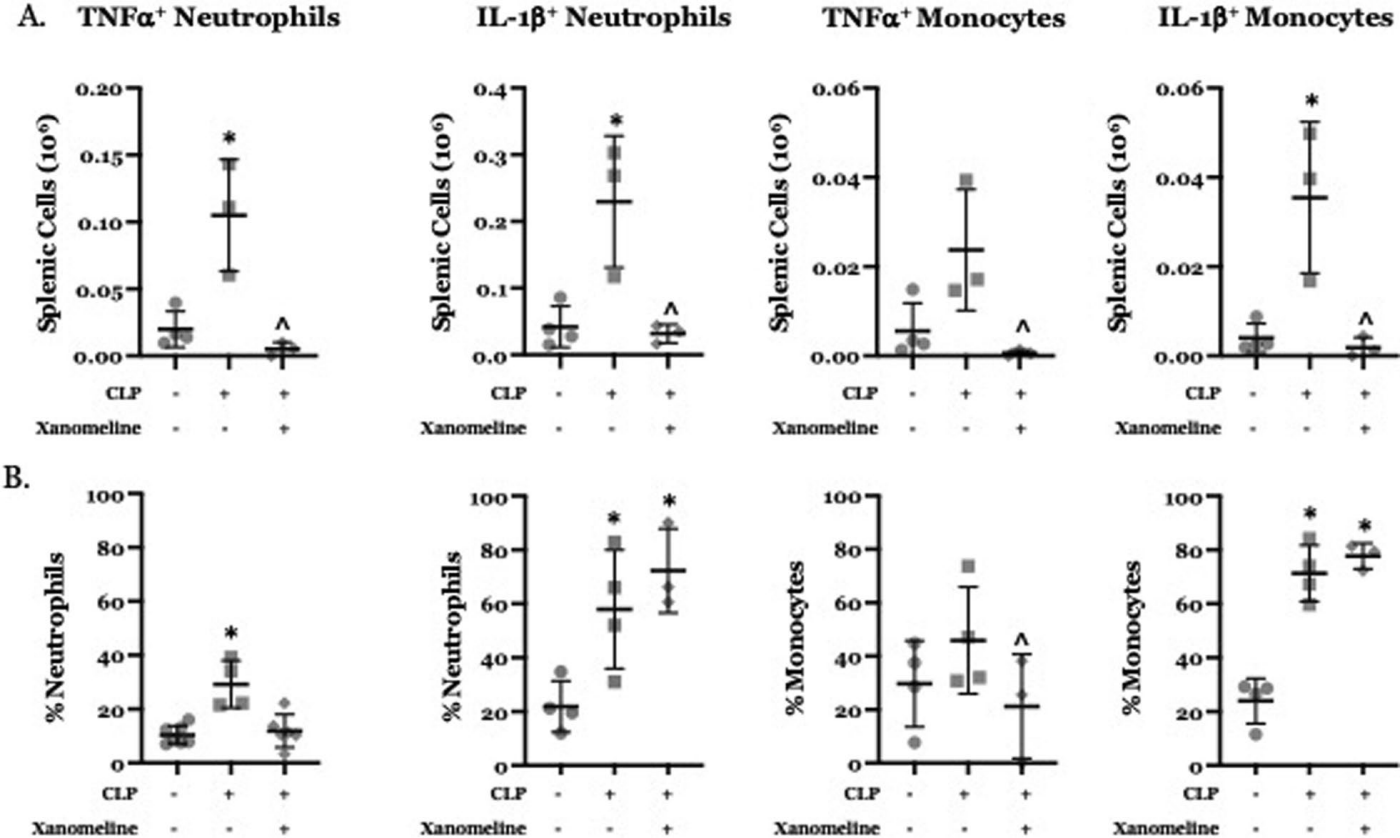
Effects of CLP and CLP + xanomeline treatment on innate immune splenic cells expression of TNFα and/or IL-1β. C57Bl6 mice, euthanized 48 h. post CLP. Cell types/cytokines identified using flow cytometry. Mean value for each animal indicated by closed circle (T_0_/baseline), closed square (48 h. post CLP) or closed diamond (xanomeline treatment of mice studied 48 Hrs. post-CLP). Long horizontal lines—mean value; error bars—± standard deviation. Significance determined using one-way ANOVA with Tukey–Kramer correction, P < 0.05. *= significantly different from value at T_0_; ^ = significantly different from value at 48 h. post-CLP without xanomeline. N = 3–7. **A** Numbers of Neutrophils and Monocytes that expressed TNFα or IL-1β in response to ex vivo stimulation with LPS. **B** Percentages of Neutrophils and Monocytes that expressed TNFα or IL-1β in response to ex vivo stimulation with LPS

We also examined how CLP affected ex vivo responses of splenic innate immune cells to stimulation with LPS. At 48 h post-CLP, the numbers of neutrophils expressing TNFα or IL-1β and the number of monocytes expressing IL-1β was higher than at baseline (Fig. 5A). Following CLP, the percentages of TNFα-expressing neutrophils and of IL-1β-expressing neutrophils and monocytes was also higher than at T_0_ (Fig. 5B). Both the numbers and percentage of TNFα^+^ monocytes post-CLP were not statistically distinguishable from baseline values. In post-CLP mice treated with xanomeline, the numbers of neutrophils and monocytes expressing either TNFα or IL-1β were significantly lower than observed values in post-CLP animals not receiving the drug and were statistically indistinguishable from baseline (Fig. 5A, B). However, the percentages of IL-1β -expressing neutrophils and monocytes was unaffected (Fig. 5B). These findings indicate that M1AChR-mediated responses contribute to the increased number of neutrophils and monocytes and that may also affect TNFα expression by these cells. However, LPS stimulated expression of IL-1β appears to be M1AChR independent.

## Discussion

Elevated serum cytokine levels following an inflammatory stimulus in part reflect decreased activity in the vagus-mediated anti-inflammatory reflex (Pavlov and Tracey 2012). Among the affected components of this pathway are cholinergic neurons in basal forebrain and splenic cells that release cytokines (Lehner et al. 2019). The central M1AChR agonist xanomeline reduced cytokine release (Rosas-Ballina et al. 2015). Studies with CLP, the most commonly-used animal model of sepsis (Osuchowski et al. 2018), indicated that reduced activity in basal forebrain cholinergic neurons contribute to inflammation and that restored M1AChR-mediated activity in the brain reduced mortality (Zhai et al. 2017; Rosas-Ballina et al. 2015). Relative to measurements at baseline (T_0_), data from 48 h. post CLP demonstrated (1) lower activity in basal forebrain cholinergic neurons and in hippocampal cells, (2) higher serum levels of TNFα, IL-1β, MIP-1α, IL-6, KC and G-CSF, (3) a larger number of splenic macrophages and inflammatory monocytes, but fewer splenic dendritic cells, CD4^+^ and CD8^+^ T cells and (4) a higher percentage of neutrophils that responded to ex vivo stimulation by secreting TNFα, and of both neutrophils and monocytes that responded by elaborating IL-1β. When CLP was followed by administration of xanomeline, differences with T_0_ in neuronal activity, serum levels of TNFα, IL-1β and MIP-1α, and number of macrophages and inflammatory monocytes were not present. However, concentrations of IL-6, KC and G-CSF and percentages on cytokine-expressing neutrophils and monocytes were higher than baseline and indistinguishable from measurement made in post-CLP mice not receiving the drug. These findings indicate that decreased M1AChR-mediated activity contributes to some, but not all, of the changes in cytokines levels and leukocyte abundance induced by CLP.

Acute encephalopathy/delirium and cognitive dysfunction in long-term survivors, both well-documented and well-described, are the generally recognized manifestations of sepsis-induced dysfunction in the brain (Girard et al. 2018; Li et al. 2022; Rengel et al. 2019). However, the contribution of the brain to the pathogenesis of organ dysfunction, the defining characteristic of sepsis, has been under-appreciated. The ability of complex organisms to respond to constant fluctuations in either the internal or external environment depends on communication and coordinated activity between cells and organs that are not in direct physical contact. Some years ago, Godin and Buchman proposed that the dysregulated host response of sepsis compromises these interactions, causing an “uncoupling of biologic oscillators” that reduced biologic variability and ultimately contributed to organ dysfunction (Godin and Buchman 1996). Organ-to-organ interactions in humans are mediated by the immune, endocrine and neuronal systems. While sepsis-induced dysfunction in the first two has been extensively examined (Boomer et al. 2011; Berghe et al. 2022), the brain has been less-well scrutinized. Nerves can disseminate signals far more rapidly than the other two systems and thus may be the “first responders” to perturbations. Indeed, neuronal activity contributes to both endocrine and immune responses, and these responses are compromised in sepsis (Deutschman et al. 2013; Berghe et al. 2022; Jakob et al. 2020). Thus, it is logical to postulate that neuronal dysfunction is a primary determinant of sepsis-induced organ dysfunction. The data presented here support that hypothesis.

The ability of xanomeline to affect some, but not all, of the differences in cytokine expression and leukocyte abundance/activity is important. Previous work has identified CLP-induced circulating levels of TNFα, IL-1β and IL-6 that are higher than T_0_ levels. Much of those data were generated while exploring the vagus-mediated anti-inflammatory reflex, a neural pathway by which central M1AChR-expressing neurons alter cytokine expression and inflammatory cell abundance in the spleen (Zhai et al. 2017; Munyaka et al. 2014; Rosas-Ballina et al. 2015). The more distal portions of this pathway involve several different neurotransmitters (Huston et al. 2009; Kressel et al. 2020; Lu et al. 2014; Rosas-Ballina et al. 2011). In effect, this pathway has been described as a “brake on inflammation” whose therapeutic potential is currently being explored (Chavan et al. 2017; Kelly et al. 2022). The findings reported here suggest that this hypothesis be viewed with a degree of caution. Pre-treatment with xanomeline attenuated LPS-induced elevations of TNFα, IL-6, IFNγ and IL12p70 while optogenetic activation of basal forebrain cholinergic neurons has a similar effect on increases in TNFα, IL-6 and IL-10 levels at a very early post-CLP timepoint (Zhai et al. 2017; Rosas-Ballina et al. 2015). However, in our studies, which examined mice 48 h. post-CLP, levels of IL-6, as well as the chemokine KC and the neutrophil growth factor G-CSF, were not affected by xanomeline. These findings suggest that some aspects of the inflammatory reflex do not involve an M1-mediated ACh pathway. Perhaps other muscarinic or nicotinic isoforms contribute (Yeomans 2012; Wills and Kenny 2021; Mineur et al. 2023; Fontana et al. 2023). Alternatively, the CLP-induced difference in IL-6 may be mediated by a non-cholinergic neural pathway. We have demonstrated CLP-induced decreases in the orexinergic system of the hypothalamus (Deutschman et al. 2013). Glutaminergic and GABAergic systems have been implicated in neuroinflammation (Zhang et al. 2023). Further investigation is clearly indicated.

Previous work also indicated that pre-treatment with xanomeline attenuated LPS induced increases in splenic dendritic cell numbers (Rosas-Ballina et al. 2015), a finding consistent with our observations post-CLP. However, administering the drug prior to LPS challenge increased numbers of splenic T cells and did not affect neutrophil or monocyte/macrophage abundance (Rosas-Ballina et al. 2015). In contrast, we found that, while the abundance of neutrophils, macrophages, and inflammatory monocytes was higher post-CLP than at baseline, levels following the addition of xanomeline were significantly lower than in untreated mice and were indistinguishable from baseline. The differing effects of xanomeline on cytokines may well reflect the timing of administration—treatment before LPS administration but after CLP. However, the discrepancies in numbers of splenic neutrophils, monocytes/macrophages and T cells suggest that mechanisms underlying some of the effects of CLP and LPS differ from each other. Importantly, CLP increased numbers of TNFα- and IL-1β-expressing neutrophils and IL-1β-expressing monocytes, differences that were eliminated when xanomeline was added. The percentages of cytokine producing cells was not altered by CLP + xanomeline. Thus, xanomeline did not appear to influence the effects of CLP on TNFα/IL-1β expression by innate immune cells, but rather simply reduced the total number of cells present.

That CLP and administration of LPS induce distinct cytokine and immune phenotypes also has implications that may affect our understanding of the pathobiology of sepsis. The data presented here and results generated by other investigators suggest that both CLP and human sepsis evoke unique immune profiles. Some of these changes can be considered “pro-inflammatory” (Taylor et al. 2020c), others “anti-inflammatory” (Boomer et al. 2011; Hotchkiss et al. 2001) but the overall response defies easy classification (Murphey et al. 2004). Indeed, the work here demonstrated that CLP-induced changes in serum levels of two quintessential “pro-inflammatory” cytokines—TNFα and IL-6—respond differently to M1AChR stimulation. Further, while elevated levels of splenic macrophages and monocytes point to an enhanced immune response, decreased numbers of dendritic cells and of CD4^+^ and CD8^+^ T cells suggest immune suppression. These discrepant findings suggest that CLP, and perhaps sepsis, involve complex and likely unique mechanisms that are not easily characterized.

This study has limitations and is subject to certain caveats. We examined responses only at 48 h. post-CLP. While previous work suggests that this approach captures most post-CLP pathobiology (Abraham et al. 2018; Deutschman et al. 2013), the dynamic nature of both CLP and sepsis mandates that investigation of the mechanisms that produce organ dysfunction be examined at additional time points.

Perhaps the most important limitation lies in attempts to model sepsis, a distinctly human disorder, using animals. While it is the most commonly-used animal model of sepsis, the deficiencies of CLP are well-documented and indisputable. The concerns include differences in gene expression and in organ-specific pathophysiologic responses to inflammation (Seok et al. 2013). Importantly, lab mice are immunologically naïve while humans are exposed to an antigenically rich environment from an early age on. These discrepancies may have limited translation of approaches to treatment; therapy that has worked in post-CLP mice has not improved outcome in human sepsis. However, when the focus is directed towards organ dysfunction, the defining clinical characteristic of human sepsis (Singer et al. 2016), some of the distinctions blur. Inflammation in mice causes hypothermia, bradycardia and hypopnea; in humans the response is characterized by fever, tachycardia and tachypnea. But both mice and men respond to inflammation with alterations in metabolism, cardiac function and respiration. Recent modifications have improved the fidelity of the model to the disorder (Rincon et al. 2021); these include our demonstration that induction of a broad memory T cell repertoire in mice prior to CLP increased organ dysfunction and reduced survival to levels closer to those observed in human sepsis (Taylor et al. 2020c). Further research is required to identify pathobiology and to enhance clinical relevance.

## Conclusions

In summary, our findings indicate that, at 48 h. post-CLP.

1. activity in hippocampal cells and in basal forebrain neurons expressing ChAT is lower than at baseline. Lower hippocampal activity reflected, at least in part, reduced activity in neurons that respond to M1AChR-mediated stimulation.
2. circulating levels of TNFα, IL-1β, MIP-1α, IL-6, KC and G-CSF were higher than at baseline. The levels of TNFα, IL-1β and MIP-1α were driven, in part, by lower levels of M1AChR stimulation. In contrast, levels of IL-6, KC and G-CSF were not affected by differences in M1-mediated activity.
3. the numbers of splenic macrophages and inflammatory monocytes were higher than at baseline and a greater percentage of neutrophils and monocytes responded to an ex vivo inflammatory stimulus. This increase resulted, in part, from lower levels of M1AChR-mediated stimulation.
4. a greater fraction of splenic neutrophils and monocytes responded to ex vivo stimulation by elaborating cytokines. This difference was not driven by lower activation of M1AChRs.
5. the spleen contained fewer central dendritic cells, and fewer CD4 + and CD8 + T cells. This difference did not reflect altered M1AChR-mediated activity.

This study re-affirms that altered activity in the brain contributes to CLP-mediated immune responses. Our findings support the hypothesis that altered activity in the brain constitutes one component of the “dysregulated host response” that is a defining characteristic of sepsis.

### Supplementary Information

**Additional file 1: Figure S1.** Gating strategies for flow cytometry. A. Gating strategy for identifying T cell sub populations. B. Gating strategy for identifying splenic innate immune cell populations shown at baseline (red), 48 h. post-CLP (blue) and 48 h. post CLP + xanomeline (yellow). **Figure S2.** Q-Q (above) and Residual Plots (below). A Cytokines. B. Splenic cells. C Cytokine-expressing Splenic cells.

**Additional file 2: Table S1.** Antibodies used for Flow Cytometry.

## Data Availability

The datasets used and analyzed during the current study are available from the corresponding author on reasonable request.
