# Supplementary figures and images for: M1 cholinergic signaling in the brain modulates cytokine levels and splenic cell sub-phenotypes following cecal ligation and puncture
Source: Mol Med. 2024 Feb 5;30:22. doi: 10.1186/s10020-024-00787-x (PMC10845657; doi:10.1186/s10020-024-00787-x)

Fig. S1


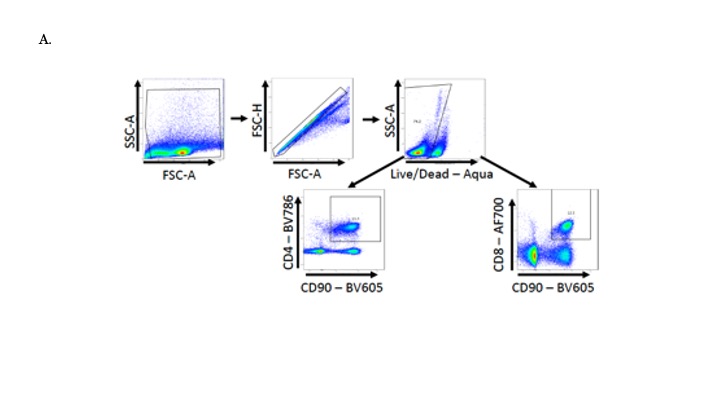


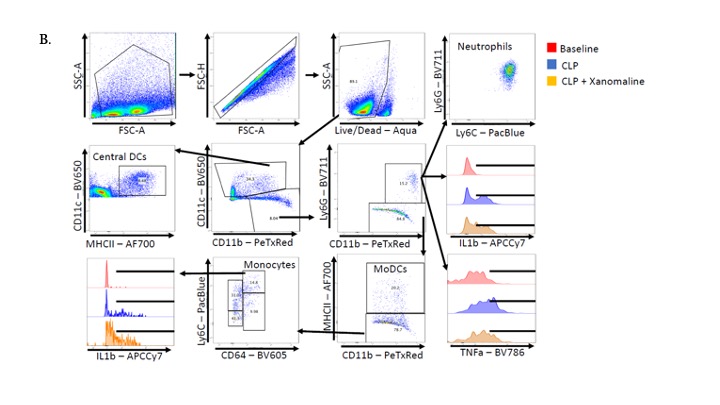


Fig. S2


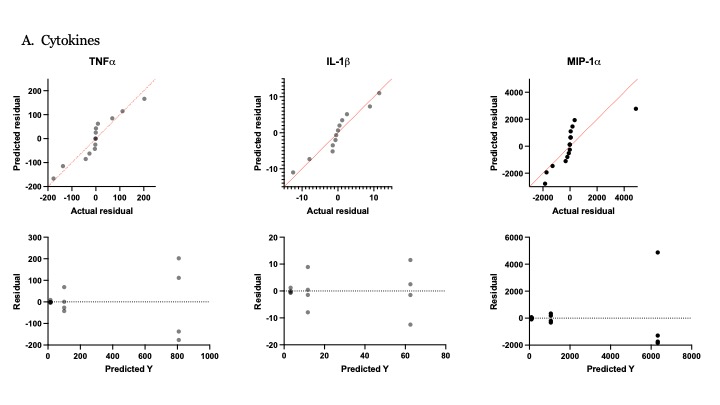


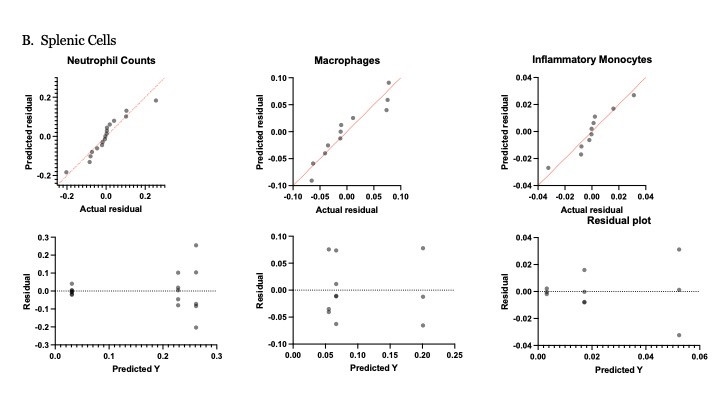


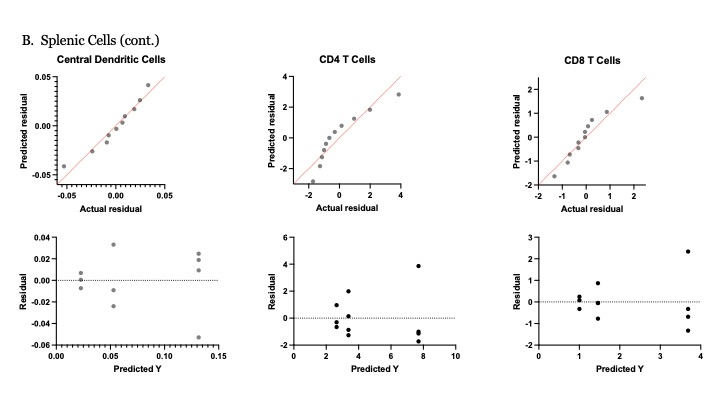


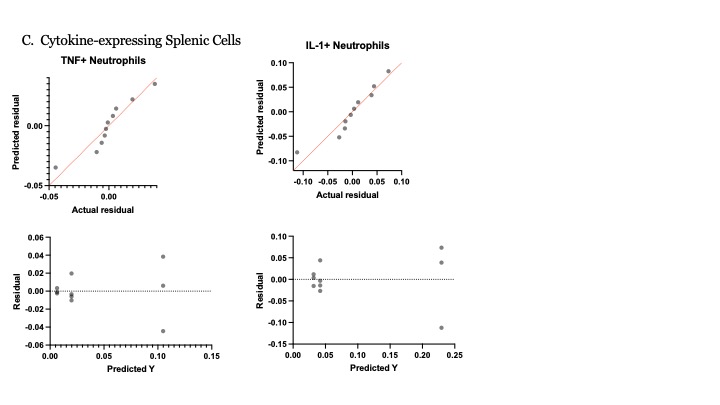


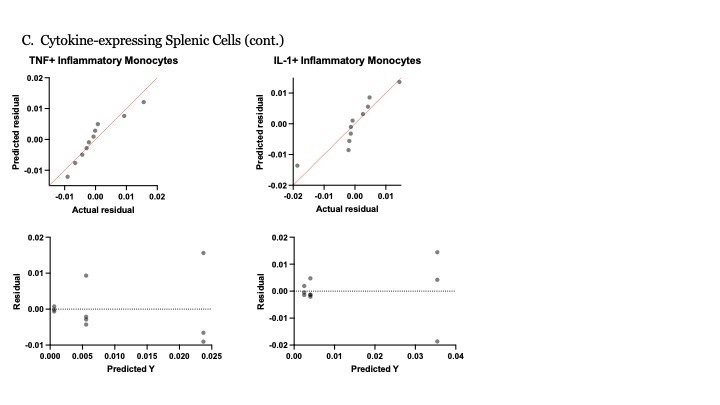

Supplement: Supplementary file 1 — Additional file 1: Figure S1. Gating strategies for flow cytometry. A. Gating strategy for identifying T cell sub populations. B. Gating strategy for identifying splenic innate immune cell populations shown at baseline (red), 48 h. post-CLP (blue) and 48 h. post CLP + xanomeline (yellow). Figure S2. Q-Q (above) and Residual Plots (below). A Cytokines. B. Splenic cells. C Cytokine-expressing Splenic cells. [file 10020_2024_787_MOESM1_ESM.docx]
