# Supplementary material for: M1 cholinergic signaling in the brain modulates cytokine levels and splenic cell sub-phenotypes following cecal ligation and puncture
Source: Mol Med. 2024 Feb 5;30:22. doi: 10.1186/s10020-024-00787-x (PMC10845657; doi:10.1186/s10020-024-00787-x)
Supplement: Supplementary file 2 — Additional file 2: Table S1. Antibodies used for Flow Cytometry. [file 10020_2024_787_MOESM2_ESM.pptx]

## Slide 1
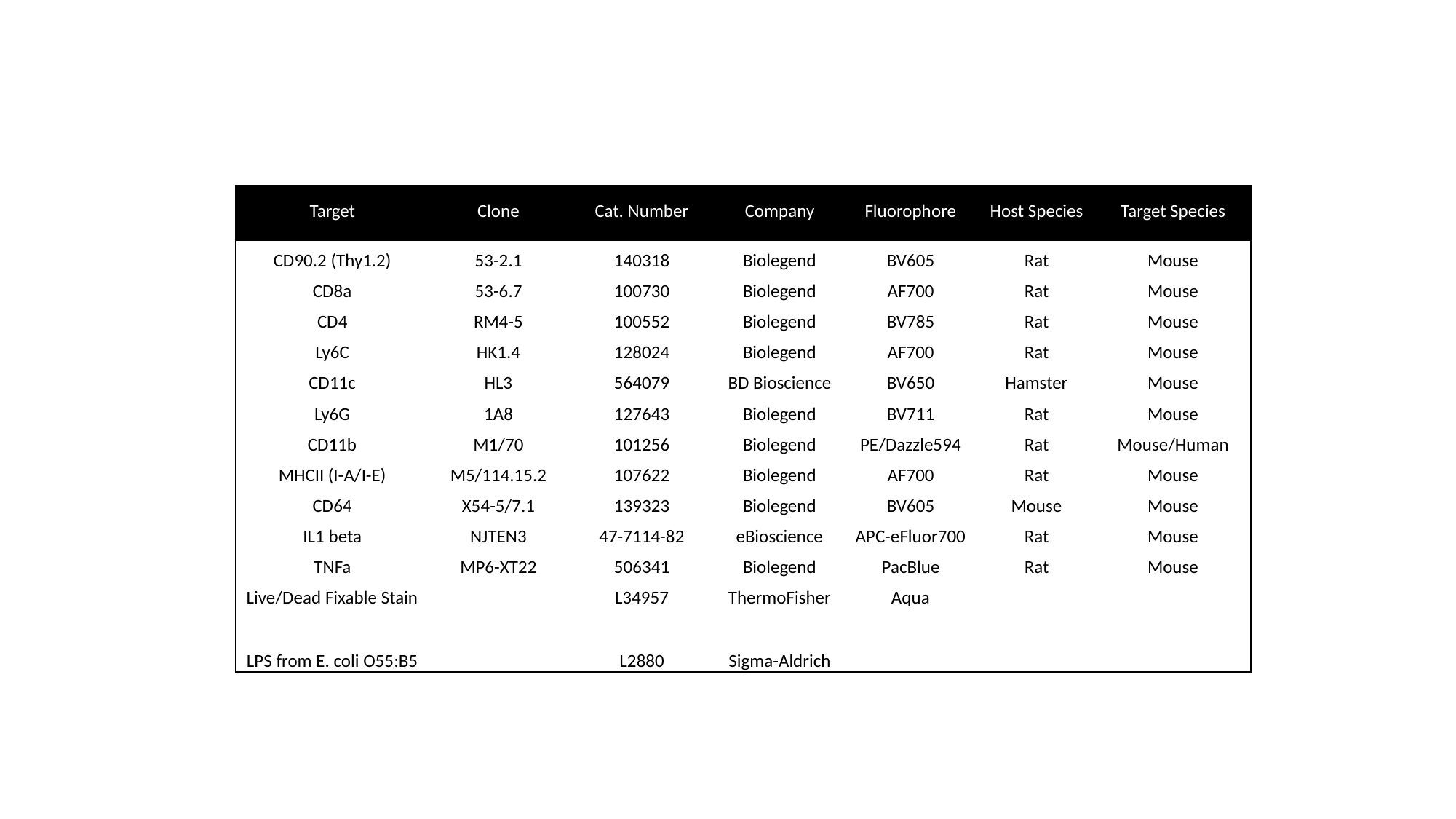

| Target | Clone | Cat. Number | Company | Fluorophore | Host Species | Target Species |
| --- | --- | --- | --- | --- | --- | --- |
| CD90.2 (Thy1.2) | 53-2.1 | 140318 | Biolegend | BV605 | Rat | Mouse |
| CD8a | 53-6.7 | 100730 | Biolegend | AF700 | Rat | Mouse |
| CD4 | RM4-5 | 100552 | Biolegend | BV785 | Rat | Mouse |
| Ly6C | HK1.4 | 128024 | Biolegend | AF700 | Rat | Mouse |
| CD11c | HL3 | 564079 | BD Bioscience | BV650 | Hamster | Mouse |
| Ly6G | 1A8 | 127643 | Biolegend | BV711 | Rat | Mouse |
| CD11b | M1/70 | 101256 | Biolegend | PE/Dazzle594 | Rat | Mouse/Human |
| MHCII (I-A/I-E) | M5/114.15.2 | 107622 | Biolegend | AF700 | Rat | Mouse |
| CD64 | X54-5/7.1 | 139323 | Biolegend | BV605 | Mouse | Mouse |
| IL1 beta | NJTEN3 | 47-7114-82 | eBioscience | APC-eFluor700 | Rat | Mouse |
| TNFa | MP6-XT22 | 506341 | Biolegend | PacBlue | Rat | Mouse |
| Live/Dead Fixable Stain | | L34957 | ThermoFisher | Aqua | | |
| | | | | | | |
| LPS from E. coli O55:B5 | | L2880 | Sigma-Aldrich | | | |
